# Long‐term oxygen treatment need is less frequent in eosinophilic COPD patients

**DOI:** 10.1111/crj.13451

**Published:** 2021-10-26

**Authors:** Nilüfer Aylin Acet‐Öztürk, Asli G. Dilektasli, Özge Aydın‐Güçlü, Ezgi Demirdöğen, Funda Coşkun, Ahmet Ursavaş, Mehmet Karadağ, Esra Uzaslan

**Affiliations:** ^1^ Faculty of Medicine, Department of Pulmonology Uludağ University Bursa Turkey

**Keywords:** COPD, eosinophil, inflammation, supplemental oxygen

## Abstract

**Introduction:**

Eosinophilic airway inflammation is a recognized inflammatory pattern in subgroups of patients with chronic obstructive pulmonary disease (COPD). However, there are still conflicting results between various studies concerning the effect of eosinophils in COPD patients. Our aim with this study was to evaluate eosinophilic inflammation and its relation to the clinical characteristics in a group of COPD patients.

**Methods:**

Stable COPD patients with FEV_1_% predicted < 50 or with ≥ 1 exacerbation leading to hospital admission or ≥2 moderate or severe exacerbation history were consecutively enrolled from outpatient clinics.

**Results:**

We included 90 male COPD patients, with a mean age of 63.3 ± 9.2. Mean FEV_1_% predicted was 35.9 ± 11.3. Eosinophilic inflammation (eosinophil percentage ≥2%) was evident in 54 (60%) of the patients. Participants with eosinophilic inflammation were significantly older and had better FEV_1_ predicted % values. Eosinophilic COPD patients were characterized with better quality of life and fewer symptoms. COPD patients with noneosinophilic inflammation used supplemental long‐term oxygen therapy (LTOT) more frequently compared to patients with eosinophilic inflammation (36.1% vs. 14.8%, *p* = 0.01). Eosinophilic inflammation is associated with less dyspnea severity measured by mMRC (OR: 0.542 95% CI: 0.342–0.859, *p* = 0.009) and less LTOT use (OR: 0.334 95% CI: 0.115–0.968, *p* = 0.04) regardless of age, severity of airflow limitation, and having frequent exacerbation phenotype.

**Conclusion:**

Our study supports the growing evidence for a potential role of eosinophilic inflammation phenotype in COPD with distinctive clinical characteristics. Eosinophilic inflammation is inversely associated with dyspnea severity measured by mMRC and LTOT use independently from age, total number of exacerbations, St. George Respiratory Questionnaire (SGRQ) total score and FEV_1_% predicted.

## 1 INTRODUCTION

Eosinophilic airway inflammation is a well‐recognized inflammatory pattern in subgroups of patients with chronic obstructive pulmonary disease (COPD). Most frequently used methodology to identify eosinophilic inflammation in airway diseases is sputum eosinophil percentage.^1^, ^2^ However, recent studies revealed a strong correlation between the peripheral blood eosinophil count and sputum eosinophils exacerbations in stable COPD^3^. In addition, a blood eosinophil count of 2% has 90% sensitivity and 60% specificity for detecting sputum eosinophilia of 3% in COPD.^4^

Definition of eosinophilic inflammation in stable COPD patients varies between studies, and there is no consensus on the definition of blood eosinophilic inflammation in the literature yet. Sputum eosinophilia has been observed in approximately 1/3 of stable COPD patients,^3^ while persistent and intermittent blood eosinophilia is seen in 37.4% and 49% of cases, respectively.^5^ Recent studies showed that blood eosinophil counts during stable COPD did not change within 1 month^6^ and demonstrated a reasonable repeatability and stability during a 12 month follow‐up^7^.

During acute exacerbation of COPD (AECOPD), reports show that eosinophilic airway inflammation is identified by blood eosinophils in 9.6%–45% of cases^8^, ^9^, ^10^, ^11^, ^12^ and by sputum eosinophilia in 28% of cases.^4^ Eosinophilic inflammation in AECOPD is associated with a shorter hospital stay,^12^, ^13^ higher COPD‐related readmission rate, and a shorter time to the first COPD‐related hospital readmission.^10^, ^14^, ^15^, ^16^, ^17^ Noneosinophilic inflammation in AECOPD that require intensive care hospitalization is associated with a prolonged ICU stay and NIMV failure.^7^

Emphysema % shown in computed tomography (CT) at baseline^5^ and self‐reported emphysema^18^ is not different between eosinophilic and noneosinophilic patients; however, emphysema progression during follow‐up is greater in noneosinophilic COPD patients.^5^ In contrast with these studies Hastie et al. showed that while blood eosinophil count is not related with emphysema, patients with sputum eosinophilia greater than 1.25% had higher emphysema indices, air trapping, and functional small airway disease.^19^ Long‐term oxygen therapy (LTOT) use^7^, ^10^ and oxygen saturation^5^, ^12^ also indicate no difference between the two inflammation groups.

Emerging evidence shows a potential role for eosinophilic inflammation indicating distinctive clinical features in COPD and a potential marker for tailored treatment approach.^20^, ^21^, ^22^, ^23^, ^24^, ^25^, ^26^, ^27^ Our aim in this study was to evaluate eosinophilic inflammation and its' relation to the clinical characteristics of COPD patients such as exacerbation history, LTOT, and domiciliary noninvasive mechanical ventilation (NIMV).

## 2 MATERIALS AND METHODS

### 2.1 Study setting and study population

Designed as a cross‐sectional study based on two pulmonary medicine departments, (Uludağ University Faculty Hospital Department of Pulmonary Diseases and Turkan Akyol Chest Diseases Hospital) outpatient clinic in between February 2016 to February 2017. We consecutively enrolled stable COPD patients with (1) FEV_1_% pred <50 or (2) ≥ 1 exacerbation leading to hospital admission, or (3) ≥2 moderate or severe exacerbation history. The institutional ethical committee approved the study protocol, and written informed consent was obtained from all participants. Exclusion criteria comprised concomitant asthma, lung malignancy, or exacerbation within 1 month.

### 2.2 Definitions

COPD patients were defined as those with chronic airway inflammation symptoms, history of exposure to risk factors, and fixed airflow limitation in spirometry.^28^ Airflow limitation was confirmed by postbronchodilator FEV_1_/FVC < 0.70. Airflow limitation was classified according to FEV_1_% predicted: >80% were defined as GOLD 1, while FEV_1_% predicted between 80 and 50, between 50 and 30, and below 30 were defined as GOLD 2, GOLD 3, and GOLD 4, respectively. Stable COPD was characterized as requiring no increase in bronchodilator use, no use of oral corticosteroids or antibiotics, no unscheduled doctor visits, or hospitalization over the previous 4 weeks.

### 2.3 Definition of AECOPD

AECOPD was defined as an acute event characterized by a worsening of the patient's respiratory symptoms beyond normal variation and leading to a change in medication, while frequent exacerbators were defined as patients experiencing two or more exacerbations per year.^28^

### 2.4 Definition of eosinophilic COPD

Patients with blood eosinophil percentage ≥2% were defined as eosinophilic COPD patients. The cut‐off value for eosinophilic inflammation was defined as 2% because of reported high correlation rates with sputum eosinophilia^3^ and the previous reports showing potential clinical relevance.^5^

### 2.5 Measurements

A detailed, structured questionnaire incorporating risk factors, smoking status, disease history, and comorbid conditions was completed for each patient. Exacerbation history was obtained from both medical records and the patients' declarations. LTOT use and domiciliary NIMV were recorded according to patient's declaration. Dyspnea severity was assessed using the modified Medical Research Council (mMRC) Dyspnea Scale and health‐related quality of life was assessed with St. George Respiratory Questionnaire (SGRQ).^29^, ^30^ SGRQ and mMRC scales were validated in Turkish.^31^, ^32^ Spirometry was performed before and after administration of short‐acting β2‐agonist (albuterol) by using Vmax Encore System (Sensormedics, Viasys, Yorba Linda, CA, USA) in accordance with ATS/ERS recommendations.^33^ Venous blood samples were taken from every patient for obtaining a total blood count.

### 2.6 Statistical analyses

Data were analyzed using Statistical Package for Social Sciences (SPPS) version 22. Means and standard deviations were reported for normally distributed continuous data and medians and interquartile ranges (ICR) for nonnormally distributed continuous data. Exacerbation rates and hospitalization within previous year were reported according to one sample poisson distribution test. Correlations between nonnormally distributed data were calculated by the Spearman test. Comparison of the two groups with nonnormally and normally distributed data was analyzed with the Mann–Whitney U test and the Student *t*‐test, respectively. Candidate risk factors related with eosinophilic inflammation were evaluated firstly by univariate analysis and then possible risk factors with *p* values below 0.10 were evaluated by multiple logistic regression model to identify independent predictors of eosinophilic inflammation. Values of *p* < 0.05 were considered statistically significant.

## 3 RESULTS

We enrolled 90 male stable COPD patients, with a mean age of 63.3 ± 9.2 years old. Mean FEV_1_ predicted % was 35.9 ± 11.3. By GOLD spirometric classification 86.6% of the patients were GOLD III or IV (Table 1). About 33.3% of the group were frequent exacerbators. About 12.2% were using domiciliary NIV, and 23.3% were on LTOT. Clinical characteristics of study group are presented in Table 1.

**TABLE 1:** Population characteristics (*n* = 90)

Age		63.3 ± 9.2
Smoking status		
Current smokers, *n* (%)		17 (18.9)
Ex‐smokers, *n* (%)		73 (81.1)
Number of comorbidities		1.0 [0.0–2.0]
*AECOPD history*		
Number of AECOPD requiring hospitalization in the last 12 months		0.0 [0.0–1.0]
Number of AECOPD requiring emergency admissions in the last 12 months		0.0 [0.0–1.0]
Total number of AECOPD in the last 12 months		1.0 [0.0–2.0]
Frequent exacerbators, *n* (%)		30 (33.3)
*Pulmonary Function Tests*		
FEV_1_/FVC, %		67.2 [63–70]
FEV_1_ predicted %		35.9 ± 11.3
FVC predicted %		43.0 ± 13.9
GOLD due FEV_1_		
	GOLD 2, *n* (%)	12 (13.3)
	GOLD 3, *n* (%)	47 (52.2)
	GOLD 4, *n* (%)	31 (34.4)
Using domiciliary NIMV, *n* (%)		11 (12.2)
Using LTOT, *n* (%)		21 (23.3)
*Quality of life and dyspnea*		
SGRQ total score		42.6 ± 18.6
SGRQ symptom score		53.2 ± 20.9
SGRQ activity score		58.8 ± 22.1
SGRQ impact score		30.0 ± 18.8
mMRC score		2.0 [1.0–3.0]
*Hemogram values*		
	Leukocyte count, (x10^9^/L)	8819.1 ± 2461.9
	Neutrophil, (%)	63.3 ± 9.6
	Lymphocyte, (%)	25.7 ± 8.7
	Eosinophil, (%)	2.2 [1.3–3.1]
	Hb, (g/dl)	13.9 ± 1.5
	Platelet, (x10^9^/L)	251022.0 ± 65581.4

*Note*: Data were expressed as numbers (percentages), mean ± SD, or median (IQR).

Abbreviations: AECOPD, acute exacerbation of COPD; COPD, chronic obstructive pulmonary disease; LTOT, long‐term oxygen treatment; mMRC, modified Medical Research Council Dyspnea Scale; NIMV, noninvasive mechanical ventilation; SGRQ, St. George Respiratory Questionnaire.

Absolute number of blood eosinophil count and blood eosinophil percentage were 195 [100–265] and 2.21% [1.3%–3.1%]. Eosinophilic inflammation (defined as blood eosinophil percentage ≥2%) was evident in 54 (60%) of the patients. Participants with eosinophilic inflammation were significantly older (*p* = 0.04) and had higher FEV_1_ predicted % values (*p* = 0.03), Table 2. Eosinophilic COPD patients were characterized by better quality of life with lower SGRQ total scores (*p* = 0.03) and less dyspnea with lower mMRC (*p* = 0.009) scores. We found that LTOT use was more frequent in patients with noneosinophilic inflammation (blood eosinophil <2%) compared with patients with eosinophilic inflammation (*p* = 0.01), Table 2. On the other hand, smoking status and frequency of acute exacerbations in the last 12 months were similar between eosinophilic and noneosinophilic patients, Table 2.

**TABLE 2:** Clinical characteristics of eosinophilic and noneosinophilic COPD subgroups

		Eosinophil < %2 *n* = 36	Eosinophil ≥ %2 *n* = 54	*p*
Age		61.0 ± 7.7	64.8 ± 9.8	0.04
Current smoking status, *n* (%)		7 (19.4)	10 (18.5)	0.91
Number of comorbidities		1.0 [0.0–1.0]	1.0 [0.0–2.0]	0.33
*AECOPD history*				
Number of AECOPD requiring hospitalization in the last 12 months		0.0 [0.0–1.7]	0.0 [0.0–1.0]	0.07
Number of AECOPD requiring emergency admissions in the last 12 months		0.0 [0.0–1.7]	0.0 [0.0–1.0]	0.14
Total number of AECOPD in the last 12 months		1.0 [0.0–2.7]	0.0 [0.0–2.0]	0.07
Frequent exacerbators, *n* (%)		15 (41.6)	15 (27.7)	0.17
*Pulmonary function tests*				
FEV_1_/FVC		67.4 [62.2–69.7]	67.2 [63–70]	0.53
FEV_1_ predicted %		32.8 ± 11.1	38 ± 11.1	0.03
FVC predicted %		40.5 ± 14	44.7 ± 13.7	0.16
GOLD due FEV_1_				0.07
	GOLD 2, *n* (%)	2 (5.5)	10 (18.5)	
	GOLD 3, *n* (%)	19 (52.7)	28 (51.8)	
	GOLD 4, *n* (%)	15 (41.6)	16 (29.6)	
Using domiciliary NIMV, *n* (%)		6 (16.6)	5 (9.2)	0.33
Using LTOT, *n* (%)		13 (36.1)	8 (14.8)	0.01
*Quality of life and dyspnea*				
SGRQ total score		48.2 ± 21.1	39.2 ± 16.3	0.03
SGRQ symptom score		55.1 ± 23.7	51.9 ± 19.4	0.51
SGRQ activity score		66.1 ± 24.4	54.4 ± 19.5	0.02
SGRQ impact score		36.1 ± 20.6	26.3 ± 16.7	0.02
mMRC score		2.0 [2.0–4.0]	2.0 [1.0–3.0]	0.009
*Hemogram values*				
Leukocyte count, (x10^9^/L)		9305.0 ± 3192.2	8495.0 ± 1783.6	0.12
Neutrophil, (%)		66.3 ± 10.7	61.3 ± 8.3	0.01
Lymphocyte, (%)		24.3 ± 9.7	26.6 ± 8	0.21
Neutrophil‐lymphocyte ratio		2.80 [1.83–4.35]	2.36 [1.92–2.98]	0.17
Eosinophil, (%)		1.1 [0.6–1.6]	2.8 [2.3–4.2]	<0.001
Hb, (g/dl)		14.1 ± 1.8	13.8 ± 1.1	0.31
Platelet, (x10^9^/L)		259166.6 ± 64032.8	245592.5 ± 66629.7	0.33

*Note*: Data were expressed as numbers (percentages), mean ± SD or median [IQR].

Abbreviations: AECOPD, acute exacerbation of COPD; COPD, chronic obstructive pulmonary disease; LTOT, long‐term oxygen treatment; mMRC, modified Medical Research Council Dyspnea Scale; NIMV, noninvasive mechanical ventilation; SGRQ, St. George Respiratory Questionnaire.

We included age, total number of exacerbations, mMRC score, SGRQ total score, FEV_1_% predicted, and LTOT use in a multiple logistic regression model to identify independent predictors of eosinophilic inflammation (Table 3). Eosinophilic inflammation is inversely associated with dyspnea severity measured by mMRC (OR: 0.50 [0.30–0.83], *p* = 0.008) and LTOT use (OR: 0.29 [0.09–0.90], *p* = 0.03).

**TABLE 3:** Multivariable analysis of predictors for eosinophilic inflammation

	Univariable analysis		Multivariable analysis	
	OR (95% CI)	*p*	OR (95% CI)	*p*
Age	1.04 (0.99–1.09)	0.05	‐	‐
Total number of exacerbations	0.75 (0.57–0.98)	0.03	‐	‐
FEV_1_% pred	1.04 (1.00–1.08)	0.03	‐	‐
SGRQ total score	0.97 (0.94–0.99)	0.03	‐	‐
mMRC	0.52 (0.33–0.82)	0.005	0.50 (0.30–0.83)	0.008
LTOT	0.30 (0.11–0.84)	0.02	0.29 (0.09–0.90)	0.03

## 4 DISCUSSION

The prevalence of eosinophilic inflammation defined as >2% eosinophils is 60% in our study population consisting of COPD patents with FEV_1_% predicted <50 or with ≥1 exacerbation leading to hospital admission or ≥2 moderate or severe exacerbation history. Eosinophilic COPD patients are older and have higher FEV_1_% predicted. These patients are characterized by fewer symptoms according to mMRC scores and better quality of life defined by SGRQ scores. Noneosinophilic COPD patients needed LTOT more frequently. Other clinical measurements like smoking status, comorbidities, and number of exacerbations in the last year were not significantly different between groups.

Determination of COPD disease severity is complex and includes lung functions, measure of breathlessness, quality of life, and exacerbation frequency. Our findings concerning age, lung functions, and severity of symptoms are in accordance with the study of Singh et al., which reported on 1483 patients from the ECLIPSE cohort. Singh et al. stated that patients with persistent eosinophil ≥2% were older, mostly male, with higher FEV_1_%, and lower SGRQ and mMRC scores.^5^ Likewise, Negewo et al. showed that patients with eosinophilic inflammation had higher FEV_1_%predicted, better BODE scores, and less symptoms measured by mMRC and SGRQ.^3^ In addition to these results, eosinophilic patients had higher chemokine ligand 18, with lower club cell protein 16 and CXCL8 levels, which indicate different activated pathways.^5^ In contrast with our results Hastie et al. showed reduced FEV_1_%predicted and higher rate of exacerbations in eosinophilic COPD patients.^19^ Wu et al. included 40 112 COPD patients in stable or exacerbation phase in recent meta‐analysis to identify clinical characteristics of eosinophilic inflammation in COPD. Using cut‐off value for blood eosinophil of 2%, 54.9% of the study population were identified as eosinophilic COPD. Eosinophilic patients were male predominant, had higher BMI, and had higher ischemic heart disease. While FEV_1_%predicted did not differ between groups, lower rate of GOLD stage 1 was identified in eosinophilic group.^34^ In patient‐level meta‐analysis by Pavord et al. blood eosinophilia >2% was present in 37 of the 10 861 patients and FEV_1_%predicted did not differ between eosinophilic and noneosinophilic patients.^35^

In our study patients with noneosinophilic inflammation tended to have more frequent exacerbations leading to hospitalization and higher total number of total exacerbations. Lonergan et al. included 7220 COPD patient and had a mean 9‐year follow‐up period. The study showed that the rate of severe exacerbations was lower in high eosinophilic COPD patients, and patients with high blood neutrophil count were related with higher mortality.^36^ Similarly, number of COPD‐related in‐patient days within severe COPD patients and overall mortality for all COPD patients were greater in noneosinophilic COPD patients in the study from Finland.^37^ However, there are some studies indicating an association between elevated blood eosinophil count and exacerbations.^38^, ^39^ Vedel‐Krogh et al. calculated a 1.76‐fold increased risk of severe exacerbation related with high blood eosinophil counts.^18^ Kerkhof et al. found an elevated risk exacerbation related with eosinophils but only in ex‐smoker COPD population.^40^ This difference between studies might be result of selection of study population. Study from Viinanen et al.^37^ had similar results with our study and had a similar population (FEV_1_%predicted ≤50%). However, Vedel‐Krogh et al.^18^ and Kerkhof et al.^40^ included all COPD patients with spirometric values and excluded asthma patients but Müllerova et al.^38^ used ICD codes to select patients and did not exclude asthma patients.

In our study, eosinophilic inflammation is inversely and independently associated with long‐term supplemental oxygen need. In studies evaluating eosinophilic exacerbations, there is no statistical difference between the two groups, regarding the need for long‐term supplemental oxygen^7^, ^10^; however, in these studies, eosinophil measurements were taken during a state of exacerbation rather than in stable state, which could explain this difference.

Wells et al. followed 678 moderate–severe COPD patients for 5 years to evaluate risk factors associated with the development of resting hypoxemia and supplemental oxygen need. This study showed a negative correlation between baseline emphysema and follow‐up oxygen saturation.^41^ Singh et al. showed that emphysema progression was statistically greater in noneosinophilic COPD patients.^5^ Oh et al. proved that severity of emphysema was independently and negatively correlated with blood eosinophil count.^42^ Independent risk factors for air trapping measured by thoracic CT in asthmatic patients are high levels of airway neutrophils and worse airflow obstruction.^43^ Considering these evidence, we can hypothesize that one of the reasons behind LTOT need in noneosinophilic patients may be the higher emphysema progression. But further studies are needed to evaluate this relation.

### 4.1 Limitations

Our study population included only male patients with FEV_1_% predicted <50 or with ≥1 exacerbation leading to hospital admission or ≥2 moderate or severe exacerbation history which is a constraint on generalizations. There is also missing data regarding possible risk factors for long‐term supplemental oxygen need; known risk factors such as heart failure, emphysema, and pulmonary artery enlargement^34^ were not assessed.

## 5 CONCLUSION

Our study supports the growing evidence of eosinophilic inflammation phenotype in COPD with differentiable clinical characteristics. Eosinophilic inflammation is inversely associated with dyspnea severity measured by mMRC and LTOT use independently from age, total number of exacerbations, SGRQ total score, and FEV_1_% predicted.

## ETHICS STATEMENT

The institutional ethical committee approved the study protocol (number: 2015‐17/21), and written informed consent was obtained from all participants.

## AUTHOR CONTRIBUTIONS

Nilüfer Aylin Acet Öztürk designed research/study, “performed research/study collected data, analyzed data, and wrote the paper. Aslı Görek Dilektaşli designed research/study, “performed research/study, collected data, analyzed data, and wrote the paper. Özge Aydin Güçlü designed research/study, “performed research/study collected data, and wrote the paper. Ezgi Demirdöğen designed research/study, contributed important reagents, analyzed data, and wrote the paper. Funda Coşkun designed research/study, contributed important reagents, analyzed data, and wrote the paper. Ahmet Ursavaş designed research/study, contributed important reagents, analyzed data, and wrote the paper. Mehmet Karadağ designed research/study, contributed important reagents, analyzed data, and wrote the paper. Esra Uzaslan designed research/study, contributed important reagents, collected data, and wrote the paper. All authors contributed in data collection and analysis. All authors have read and approved the final draft.

## FUNDING INFORMATION

Financial support was not provided.

## CONFLICT OF INTEREST

Authors have no conflict of interest regarding this paper.

## Data Availability

The data that support the findings of this study are available from the corresponding author upon reasonable request.
